# *Pseudomonas aeruginosa* as an Etiologic Agent of Nephrolithiasis in Deep Water Divers

**DOI:** 10.1089/cren.2016.0117

**Published:** 2017-01-01

**Authors:** Victoria Y. Bird, Ryan Chastain-Gross, Raymond Sutkowski, Vincent G. Bird, Paulas Vyas, Ryan Joseph

**Affiliations:** ^1^ Department of Urology, University of Florida, Gainesville, Florida.; ^2^ Department of Radiology, University of Florida, Gainesville, Florida.; ^3^ Department of Biology, College of Liberal Arts and Sciences, University of Florida, Gainesville, Florida.

**Keywords:** infection, urinary stone, diving, P-valve

## Abstract

***Background:*** A number of occupations and professions may be associated with unique hazards relevant to urologic care.

***Case Presentation:*** We relate the presentation, care, and the occupational hazard of urinary tract infection (UTI), presenting as cystitis and pyelonephritis, with stone formation in a scuba diver. The patient voiced concern that his diving suit malfunction was related to his UTI and stone disease. We review the risk of UTI in the diving environment. We also report the development of infection-related stone in this case. Our evaluation included consultation with an expert in diving and associated equipment.

***Conclusion:*** Careful installation of P-valves in dry suits, proper maintenance, and monitoring for leakage improved post-dive hygiene, and proper maintenance of P-valve tubing and diving equipment may decrease the incidence of these complications described. Urologists treating UTI and stone disease should be aware of this occupation-related hazard.

## Introduction and Background

Dry suits are commonly worn by deep water divers undertaking long exposures in cold water. The ability to urinate during such dives is facilitated by a variety of valve type devices that conduct urine from the urethra to the outside environment. Malfunction of these valves has been previously demonstrated to be a cause of urinary tract infection (UTI).^1^

Infection of the urinary tract by a number of microbial species, including Psuedomonads, has been associated with urinary stone formation. However, *Pseudomonas* infection of the urinary tract has also been associated with diving activities in fresh and sea water. *Pseudomonas* species are present in many different environments, including a variety of aquatic fresh and oceanic environments. Stone formation from occupational diving-related UTI has not been previously demonstrated or reported. We report the first case in which occupational-related UTI, resulting from valve malfunction, resulted in recurrent UTI presenting as pyelonephritis and urinary stone formation.

## Presentation of Case

A 39-year-old male professional cave diver presented to his primary care provider with dysuria and suprapubic pain. He was diagnosed with cystitis. A urine culture was performed, which revealed the presence of pansensitive *Pseudomonas aeruginosa*. After being treated for recurrent UTI with *P. aeruginosa*, and having developed gross hematuria, the patient was referred to urology for further investigation. The patient was a healthy male without significant medical or surgical history. He had no history of voiding dysfunction other than symptoms associated with UTI. Physical examination was consistent with overall good state of health. Genitourinary examination revealed normal anatomy and no significant findings. Laboratory analysis revealed UTI. Radiologic imaging, including CT, demonstrated a large right partial staghorn renal calculus. The patient was counseled as to all treatment options and he elected percutaneous nephrostolithotomy (PCNL).

**FIG. 1.**
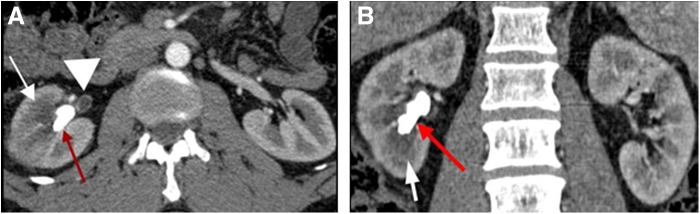
Preoperative axial **(A)** and coronal **(B)** arterial phase CT images at the level of the kidneys showing partial staghorn calculus in the right collecting system (*red arrows*). There is heterogeneous enhancement of the right kidney (*white arrows*) radiographically consistent with pyelonephritis and abnormal proximal right ureteral enhancement (*white arrowhead*) radiographically consistent with ureteritis.

**FIG. 2.**
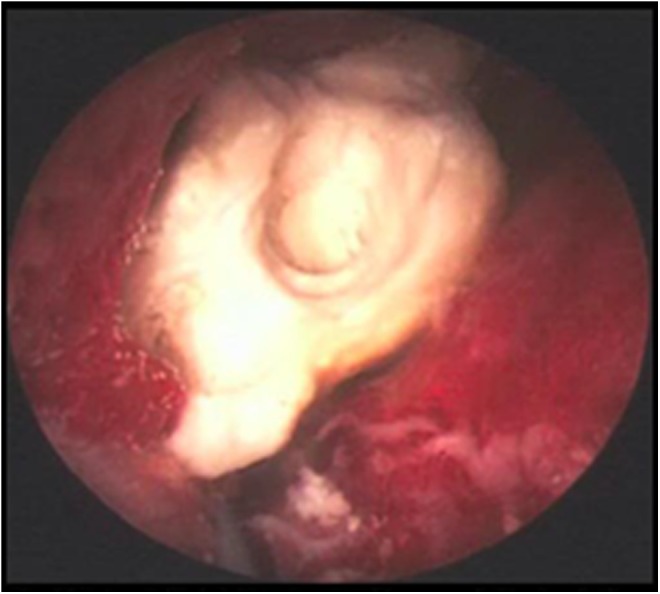
Photograph of the infected staghorn calculus. On gross examination, calculus took the form of tan-yellow, bosselated stone with loosely adherent blood clots. Crystallographic analysis (Louis C. Herring and Company) revealed calculus composition of 67% magnesium-ammonium-phosphate-hexahydrate (struvite) and 30% calcium phosphate in carbonate form, calculus culture grew *Pseudomonas aeruginosa*.

The patient underwent effective PCNL (Fig. 1). Recurrent *P. aeruginosa* UTI (both cystitis and pyelonephritis) resolved after treatment with complete stone removal and antibiotic treatment. Stone analysis demonstrated 67% struvite and 30% calcium phosphate carbonate composition (Fig. 2); stone culture grew *P. aeruginosa.* The patient recovered well from the procedure. Follow-up urine culture and radiologic imaging, CT and a subsequent surveillance ultrasonography, have since shown no evidence of UTI or recurrent urinary lithiasis.

## Discussion and Literature Review

We review the risk of UTI in the diving environment. We also report the development of infection-related stone in this case. Our evaluation included consultation with an expert in diving and associated equipment.

**FIG. 3.**
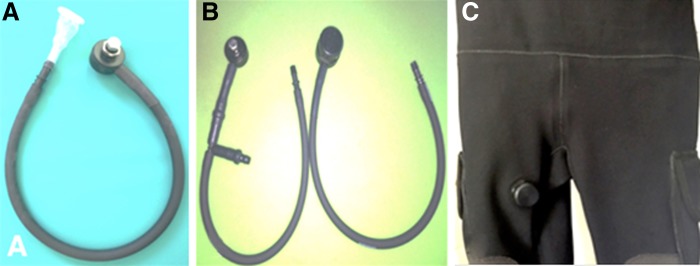
P-valves can be unbalanced **(A)** or balanced **(B)**. Balanced P-valves equalize the pressure between the valve system and the outside water pressure, avoiding reflux of water at high pressure. In addition, it prevents “genital squeeze” that can occur with an unbalanced system's tubing. An unbalanced P-valve also protects from water entering into the suit; however, it may allow some retroflux of ambient water into the P-valve tubing system at extreme depths. P-valves can be installed in the upper thigh of a dry suit **(C)**.

The patient expressed concern of diving suit malfunction related to his UTI and stone disease. We inquired into the nature of the patient's concerns about UTI and diving. He described dry suit P-valve dysfunction. While diving, the final pathway of urine to the water is through a suit bulkhead known as a P-valve (Fig. 3). It was revealed that the P-valve malfunctioned, which allowed water from the environment to retroflow into his P-valve tubing and possibly his urinary tract.

There are two types of P-valves, unbalanced and balanced. Our diver had an unbalanced P-valve. An unbalanced P-valve will allow water to retroflow into the tubing as soon as it is open, right before voiding or after voiding. A balanced P-valve will not allow outside external water to retroflow into the tubing when opening the tubing before or after voiding because of its airlocked system, thus providing an extra layer of protection from the external environment.

Use of a defective P-valve can lead to complications such as UTI (cystitis), pneumaturia, and urosepsis,^1^ as well as infected staghorn renal calculus in this case. Literature has shown that *P. aeruginosa* is commonly found in divers who develop UTIs.^1^ UTI in deep water divers is associated with *P. aeruginosa*, from fresh and sea water.^2,3^ *Pseudomonas* is a gram-negative rod-shaped bacterium with pili and flagellum that allow this bacterium to migrate to upper urinary tract. It inhabits terrestrial, aquatic (fresh and ocean water), plant, animal, and humans, and is a common bacterial cause of nosocomial infections. Upon contact with the human body, *Pseudomonas* thrives in urine because of urine osmolarity, the availability of iron, and the presence of the Tamm–Horsfall protein. Struvite or magnesium-ammonium-phosphate calculi forms in alkaline pH secondary to elevated ammonium, facilitated by urease-producing organisms, such as *Proteus* species, *Klebsiella pneumoniae*, and *P. aeruginosa.*^4^

It is possible that after multiple dives with a suit containing a defective P-valve, water and urine from previous dives may remain in the tubing. This excess urine and water serve as a growth medium for *P. aeruginosa.*^1^ As the diver continued diving without fixing the malfunction, his urinary tract was exposed to this possibly infected residual fluid, leading to the patient's recurrent UTI (cystitis and pyelonephritis) and urinary stones. This is further supported by the disappearance of his complaints after treatment and once the defects were repaired.

Female P-valves have been introduced only recently.^1^ As a result, they are generally in limited supply. Female P-valve systems work by having a cup attached to the female vaginal area. Urine is collected in a tube that connects to the cup, similarly to how a male P-valve functions.^1^ There is little to no published information about infection in female divers because the female P-valve systems are relatively new. However, because they function similarly to male P-valve systems, female divers may be expected to face similar risk to infections to that which male divers face.

## Conclusions

Catheter systems with a P-valve system in diving suits allow divers to urinate while staying underwater and avoid retroflow of ambient water and urine into the P-valve tubing system under high barometric pressure. This is the first reported case of an infectious kidney stone secondary to *P. aeruginosa*, resulting from a malfunctioning dry suit P-valve system. UTIs, including pyelonephritis, and urosepsis are a risk during deep water diving mainly because of malfunction of the P-valve.

Careful installation of P-valves in dry suits (Fig. 3), proper maintenance and monitoring for leakage, and improved postdive hygiene may decrease the incidence of these complications described. Urologists treating stone disease should be aware of this occupation-related hazard. Further investigation is needed to better discern the best type of P-valve and maintenance regimen to mitigate against UTI and possible stone formation.

## Abbreviations Used

CT: computed tomography
PCNL: percutaneous nephrostolithotomy
UTI: urinary tract infection

## References

[1] Harris R Genitourinary infection and barotrauma as complications of “P-valve” use in drysuit divers. Diving Hyperb Med 2009;39:210–212 22752741

[2] Khan N, Ishii Y, Kimata-Kino N, et al. Isolation of *Pseudomonas aeruginosa* from open ocean and comparison with freshwater, clinical, and animal isolates. Microb Ecol 2007;53:173–186 17206394 10.1007/s00248-006-9059-3

[3] Viktorov, Il'in, Polikarpov, et al. [Microbiological aspects of the environment of deep sea habitats]. Kosm Biol Aviakosm Med 1991;25:17–21 8577156

[4] Broomfield RJ, Morgan SD, Khan A, Stickler DJ Crystalline bacterial biofilm formation on urinary catheters by urease-producing urinary tract pathogens: A simple method of control. J Med Microbiol 2009;58:1367–1375 19556373 10.1099/jmm.0.012419-0

